# Manufacturing Feasibility and Forming Properties of Cu-4Sn in Selective Laser Melting

**DOI:** 10.3390/ma10040333

**Published:** 2017-03-24

**Authors:** Zhongfa Mao, David Z. Zhang, Peitang Wei, Kaifei Zhang

**Affiliations:** 1 State Key Laboratory on Mechanical Transmission, Chongqing University, Chongqing 400044, China; zhongfamao@cqu.edu.cn (Z.M.); peitangwei@cqu.edu.cn (P.W.); kaifeizhang@cqu.edu.cn (K.Z.); 2 College of Engineering, Mathematics and Physical Sciences, University of Exeter, North Park Road, Exeter EX4 4QF, UK

**Keywords:** selective laser melting, additive manufacturing, copper alloy, relative density, Vickers hardness

## Abstract

Copper alloys, combined with selective laser melting (SLM) technology, have attracted increasing attention in aerospace engineering, automobile, and medical fields. However, there are some difficulties in SLM forming owing to low laser absorption and excellent thermal conductivity. It is, therefore, necessary to explore a copper alloy in SLM. In this research, manufacturing feasibility and forming properties of Cu-4Sn in SLM were investigated through a systematic experimental approach. Single-track experiments were used to narrow down processing parameter windows. A Greco-Latin square design with orthogonal parameter arrays was employed to control forming qualities of specimens. Analysis of variance was applied to establish statistical relationships, which described the effects of different processing parameters (i.e., laser power, scanning speed, and hatch space) on relative density (RD) and Vickers hardness of specimens. It was found that Cu-4Sn specimens were successfully manufactured by SLM for the first time and both its RD and Vickers hardness were mainly determined by the laser power. The maximum value of RD exceeded 93% theoretical density and the maximum value of Vickers hardness reached 118 HV 0.3/5. The best tensile strength of 316–320 MPa is inferior to that of pressure-processed Cu-4Sn and can be improved further by reducing defects.

## 1. Introduction

Selective laser melting (SLM) is a unique additive manufacturing (AM) technology that enables us to produce dense metal parts by means of layer-by-layer construction using metal powders based on CAD models [1,2,3]. In comparison with traditional manufacturing methods, SLM offers a wide range of advantages. It can directly manufacture highly complex parts that are difficult or unable to be fabricated by conventional methods, with mechanical properties comparable to those of wrought materials. It does not require expensive tooling and machining in manufacturing and, therefore, saves manufacturing costs and lead-time [4,5]. It is also a favorable method for the fabrication of functionally-graded multi-material parts [6]. Furthermore, SLM technology offers high degrees of freedom for designers throughout the product development, from conceptual design to low-volume trial production [7]. These advantages make SLM a very useful method for the fabrication of biocompatible implants, conformal cooling channels of heat exchangers, light-weight structures in biomedicine, molds, the aerospace industry, and other fields [2,8]. However, current studies on SLM are limited and mainly focus on steel and iron-based alloys, titanium and its alloys, and Inconel and nickel-based alloys [2]. Copper alloys have recently appeared as a new type of potential material for SLM.

Copper alloys, compared with other metal alloys, exhibit moderate mechanical properties, high corrosion resistance, excellent electrical and thermal conductivity, as well as outstanding machinability and formability [9]. As such, these alloys are widely used in electronics, machinery, aerospace, defense, and other industrial fields, such as heat exchangers for various types of equipment, high-precision springs and bearings, electronic connectors, plastic deformation tools, and propulsion devices in marine applications [10,11]. However, fabrication methods of copper alloys are limited in conventional powder metallurgy technology, cold compaction, sintering, and infiltration. These methods are not appropriate for manufacturing parts with intricate shapes. Therefore, a large number of valuable studies were reported about copper alloys in direct metal laser sintering (DLMS), including balling phenomenon, densification mechanisms, and microstructural characteristics during the sintering process [12,13]. Meanwhile, the combination of design flexibility, excellent process capabilities, and the fully dense mechanism offered by SLM makes SLM a very attractive potential method for creating copper alloy components. It was reported that thin-wall W-Cu alloy components could be directly manufactured by SLM and influences of different energy input on the thickness of single-track walls were previously analyzed [14]. The research on Cu-10Sn bronze for SLM showed that its yield and ultimate strengths were much higher than those attained by casting and were accompanied by a significant improvement in ductility [11]. Moreover, multi-materials including copper alloy, such as 316L stainless steel and C18400, AlSi10Mg and C18400, were explored by SLM and the interfacial character was fully analyzed [15,16]. Cu-Cr-Zr-Ti alloy specimens were also fabricated by SLM and their microstructure and mechanical properties were investigated and compared to hot-rolled samples [17].

There are quite a few difficulties in manufacturing copper alloy parts in SLM. The high thermal conductivity and reflectivity of copper alloys at the laser result in significant heat loss and inadequate melting of powder [16]. A systematic experimental process to effectively identify appropriate SLM processing parameters for copper alloys is minimally discussed in the literature. Therefore, in order to explore the manufacturing process of copper alloys in SLM, in this work the influences of SLM processing parameters on forming properties of Cu-4Sn are investigated using experimental methods. The Cu-4Sn bronze was selected for further investigation of microstructure and mechanical properties in SLM because it has the minimum casting shrinkage coefficient among non-ferrous metals and has relatively slight dendrite segregation among tin-copper alloys, as the segregation depends on the level of tin.

## 2. Materials and Methods

### 2.1. Experimental Materials

**Figure 1:**
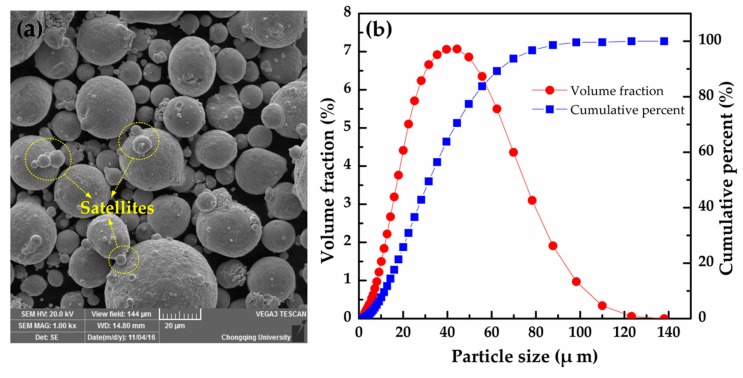
(**a**) SEM morphology and (**b**) particle size distribution of the Cu-4Sn powder.

The feedstock material used for this investigation was gas-atomized Cu-4Sn powder produced by SNDVARY (Wuxi SNDVARY New Powder Materials Technology Co., Ltd., Wuxi, China) and its nominal chemical composition (wt %) was: Cu = balance, Sn = 3.95%, impurities = 0.2%, maximum. The morphology of the powder was observed using scanning electron microscopy VEGA2 (TESCAN, Brno, Czech Republic). The raw powder is almost spherical with minor satellites, as shown in Figure 1a. Particle size distribution was measured using a laser particle size analyzer BT-9300Z (Bettersize Instruments Ltd., Dandong, China). The result in Figure 1b shows an essential log-normal distribution with particle sizes of 11.66 μm (D10), 31.81 μm (D50), and 63.36 μm (D90).

### 2.2. Experimental Equipment

Experiments were performed on the commercial SLM machine EOSINT M280 (EOS GmbH, Krailling, Germany). The source of radiation is a single-mode continuous wave ytterbium fiber laser YLR-200 (IPG Photonics, Oxford, MS, USA) operating at a wavelength of 1.07 μm and producing a laser beam with an energy intensity distribution with a Gaussian profile. The main characteristics of this machine are as follows: the maximum laser power is 195 W; the maximum laser scanning speed is 7 m/s and the laser spot size on the surface of the powder bed is 100 μm. The process chamber provides a closed environment filled by inert gas, such as nitrogen or argon, and the substrate temperature can be chosen according to different materials.

### 2.3. Experimental Methods

First, single-track experiments with different combinations of processing parameters were conducted to preliminarily narrow down selection windows during the exploration stage. Some key factors were considered as variables and the others were fixed in the experiment. The linear energy density (LED), defined by the ratio of laser power (LP) and scanning speed (SS), is a key factor in SLM [18,19]. The processing parameters used for the single-track experiments are summarized in Table 1. In order to minimize the effect of oxygen content on single tracks, it was not until the oxygen content was less than 0.1% that the argon supply to the chamber was stopped. Geometrical morphologies of single-tracks were surveyed by a Digital Microscope VHX-1000 series (Keyence, Osaka, Japan).

**Figure 2:**
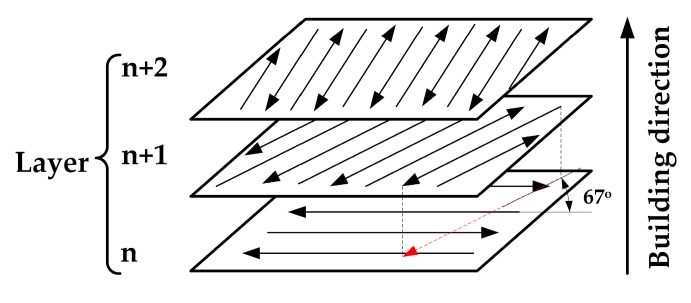
Scanning strategy used in SLM experiments.

Based on single-track experiments, a Greco-Latin square design experiment was performed for the fabrication of the Cu-4Sn bulk specimens with dimensions of 5 mm × 5 mm × 5 mm. In this experiment, the major processing parameters would be further redefined. The LP varied from 100 to 195 W with a step of 25 W; the SS varied from 100 to 300 mm/s with a step of 50 mm/s; and the hatch space (HS) varied from 100 to 180 μm with a step of 20 μm. The scanning stripe width was fixed at 3 mm and other parameters were kept the same as in the single-track experiment. The cross-hatching scanning strategy was employed whereby parallel alternative scan vectors were overlaid at an angle of 67° to the previous deposited layer, as shown in Figure 2. This scanning strategy has been proven to be an effective method of improving the surface roughness and reducing defects in SLM [20]. Densities of as-built specimens were measured by an electronic balance using the Archimedes method. Relative density (RD) is presented as a percentage of measured density and theoretical density (8.92 g/cm^3^). Vickers hardness was measured on polished cross-sections of each specimen using a hardness tester NH-5L (Shanghai EVERONE Precision Instruments Co., Ltd., Shanghai, China) with a 300 g load for 5 s. For each condition, all specimens were measured at least 10 times and results averaged after eliminating false data, so as to avoid the measuring deviation.

**Figure 3:**
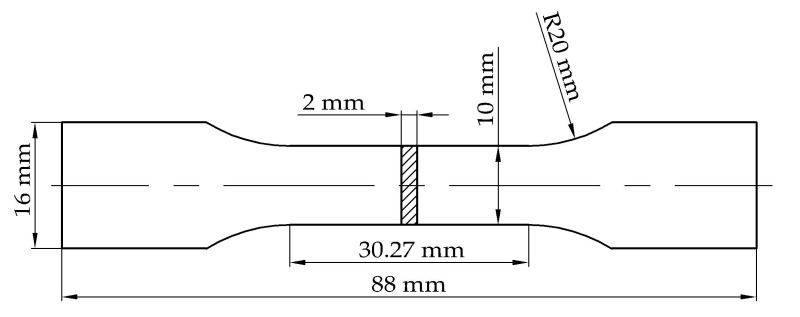
Configuration of tensile specimens.

According to the optimal processing parametric set in the Greco-Latin square design experiment, tensile specimens were fabricated and the configuration followed the Chinese GB/T228.1-2010 standard, as shown in Figure 3. Tensile strengths of these specimens were evaluated using an electronic universal tester CMT5105 (MTS, Eden Prairie, MN, USA) and fracture faces were examined by scanning electron microscopy VEGA2 (TESCAN, Brno, Czech Republic). Simultaneously, the chemical compositions were characterized by the energy dispersive spectroscopy (EDS) technique.

## 3. Results and Discussion

### 3.1. Single-Track Experiments

**Figure 4:**
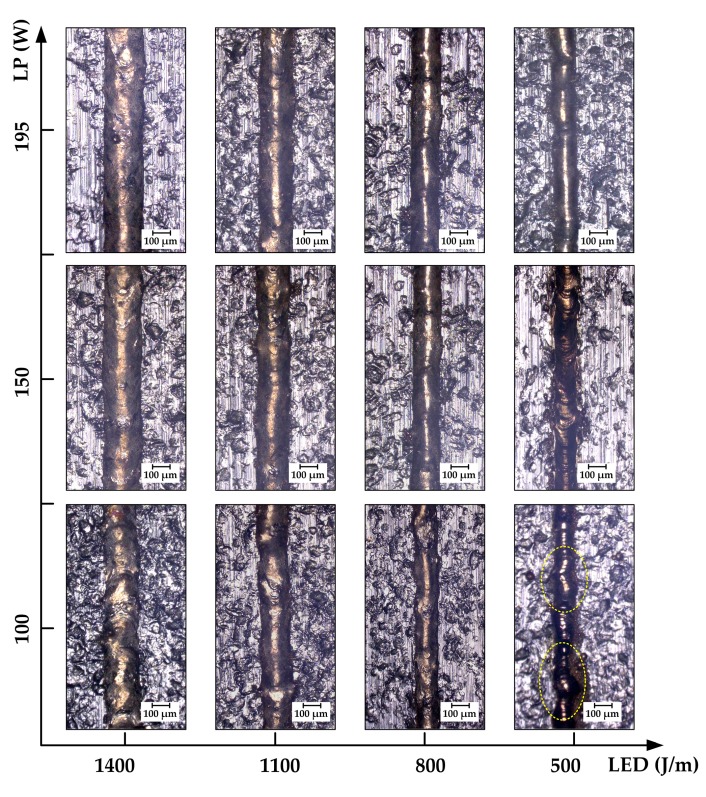
The morphologies of single tracks with different processing parameters.

Figure 4 shows morphologies of single-tracks produced by SLM on the MS1 substrate during single-track experiments. The horizontal coordinate represents LED with the values (decreasing from 1400, 1100, 800, to 500 J/m) and the vertical coordinate represents LP with the values (increasing from 100, 150, to 195 W). It can be seen from Figure 4 that solidified single-tracks present different characteristics under different processing conditions. For the same LP, the widths of single tracks decrease as the LED decreases (i.e., a decrease as the SS increases). Irregularities and distortions are observed when the LED decreases to 500 J/m, as illustrated in the inset (LED is 500 J/m, LP is 100 W) of Figure 4. This phenomenon can be explained by the fact that the less laser energy irradiated on the powders, the narrower the heat-affected zone, which can even give rise to incomplete melting of powdery material. A similar phenomenon was previously reported [21]. For the same LED in Figure 4, a smoother and more uniform surface morphology can be seen with the increase of the LP, as shown in the inset (LED is 1400 J/m, LP is 195 W). It is emphasized that the surface roughness has worsened in both low LP and high LED, such as the single track (LED is 1400 J/m, LP is 100 W), which can be attributed to the unstable molten pool where a strong Marangoni flow was formed in virtue of the decreasing SS. Based on the single-track experiments, a rough processing parameter window, high LP, and large LED can be acquired.

### 3.2. The Experiment of Greco-Latin Square Design

**Figure 5:**
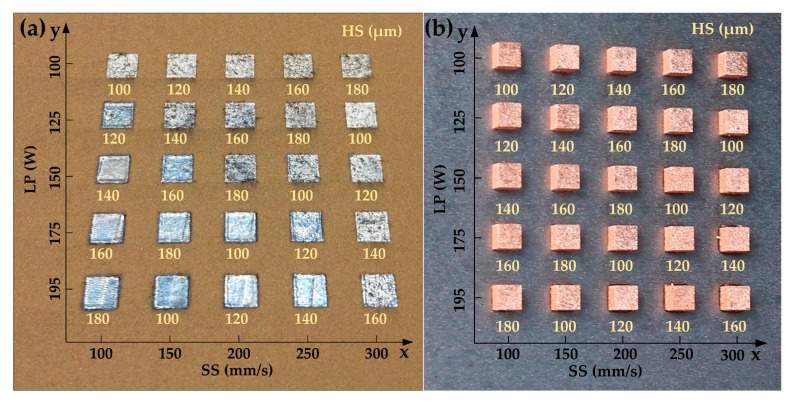
The morphologies of (**a**) single-layers and (**b**) specimens with various processing parameters.

Figure 5 shows the surface appearances of solidified Cu-4Sn single-layers and the corresponding bulk specimens, respectively. Thanks to the Greco-Latin square design used in this study, the number of experiments is effectively reduced without influencing the analysis of experimental results. The experimental orthogonal array is given in Table 2. The fabricated layers and specimens are distributed in the regular sequence whereby LP decreases along the y-axis, SS increases along the x-axis and HS arranges according to the orthogonal method.

Figure 5a reveals that qualities of single layers are significantly affected by different processing parameters. The LP, the energy intensity of the direct heat source, greatly determines the degree of melting of the present powder layer and re-melting depth of the previous layer. It shows a trend in Figure 5a that incomplete melting becomes more evident with the decrease of LP. The SS, characterizing the interactive time between the laser beam and powdery material, has a slight influence on the qualities of the single-layer. The HS represents the overlapping level of the previous track and the present track, which influences the joint strength between two adjacent single-tracks. Some black particles (i.e., incompletely melted powders) can be seen when the HS is wider than the width of a single-track, as shown in the case in Figure 5a whereby HS = 180 μm, LP = 150 W and SS = 200 mm/s. However, final properties of specimens produced by SLM depend strongly on the interaction of various parameters rather than the influence of a single factor, which can be validated hereinafter through RD values of these specimens in Figure 5b. Here, it is worth noting that the difference in surface color of the specimens between Figure 5a,b is due to different photographing environmental conditions.

The measured results of density and Vickers hardness are elaborated in Table 2. From Table 2, the maximum RD of 93.68% theoretical density is obtained in the standard order 22, with parametric combinations of LP = 195 W, SS = 150 mm/s and HS = 100 μm. Meanwhile, the maximum Vickers hardness of 118 HV 0.3/5 is acquired corresponding to the standard order 23, which is significantly higher than the traditional pressure-processed Cu-4Sn.

**Figure 6:**
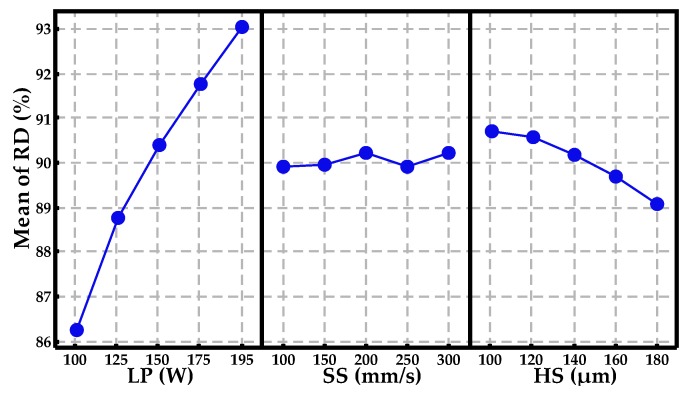
Main effects plot of processing parameters for the mean value of relative density.

**Figure 7:**
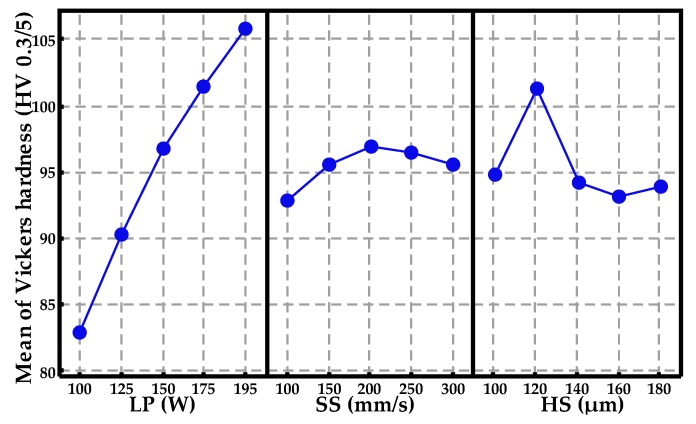
Main effects plot of processing parameters for the mean value of Vickers hardness.

The main effects plots of RD and Vickers hardness are shown in Figure 6 and Figure 7, respectively. It is clear from Figure 6 that the RD mainly depends on the LP and increases significantly as LP increases from 100 to 195 W. In contrast, the SS has few impacts on the RD and its inset illustrates a minor fluctuation at around 90% with the increase of SS from 100 to 300 mm/s. The RD has a negative correlation with the HS, which shows a relatively gentle downward tendency with the increase of HS from 100 to 180 μm. By comparing Figure 6 to Figure 7, the effect of LP on Vickers hardness presents basically the same trends as that on RD. The differences in Figure 7 are that Vickers hardness plots have peak points versus SS and HS, respectively, which means relatively optimal processing parameters of the Vickers hardness (i.e., SS = 200 mm/s and HS = 120 μm). The phenomenon can be explained by the solidification time being decreased as the SS increases, resulting in the formation of a fine crystal microstructure on the surface of specimens, while a further increase of the SS inhibits inverse segregation of the tin element and the generation of the intermetallic compounds from peritectic and eutectic reactions. The effect of HS on Vickers hardness can be attributed to thermal cycling, which has a significant influence on crystal microstructure transformation and element redistribution in the overlapping zone.

### 3.3. Analysis of Variance

In order to more accurately analyze the statistical influences of various factors, analysis of variance (ANOVA) was carried out using a general linear model based on these RD and Vickers hardness values. There are three impact factors containing LP, SS, HS, and two corresponding responses containing RD and Vickers hardness in ANOVA. Factorial information and results of ANOVA are exhibited in Table 3. The *p*-value and F-value were used to judge the significance levels of the factors. A *p*-value less than 0.05 indicates that the influence of this factor is statistically significant. Furthermore, the *p*-value less than 0.01 is termed highly statistically significant [22]. The F-value is the ratio of mean square errors from various factors and experimental errors and when it is below 1 it suggests that the effect of a factor is smaller than that of experimental error; therefore, the factor can be neglected in this experiment [23]. In Table 3, both the F-value (170.59) and *p*-value (0) of the LP in RD ANOVA prove that the LP is the most significant impact factor, followed by the F-value (10.74) and *p*-value (0.001) of the HS. It is noted that the SS has no influence on RD results because the *p*-value (0.586) is greater than 0.25 and the F-value (0.73) is below 1. The Vickers hardness ANOVA maintains a pronounced similarity with RD ANOVA. In the Vickers hardness ANOVA, the LP and the HS convey the conspicuous impacts on the Vickers hardness, and the SS also has no effect on the Vickers hardness.

**Figure 8:**
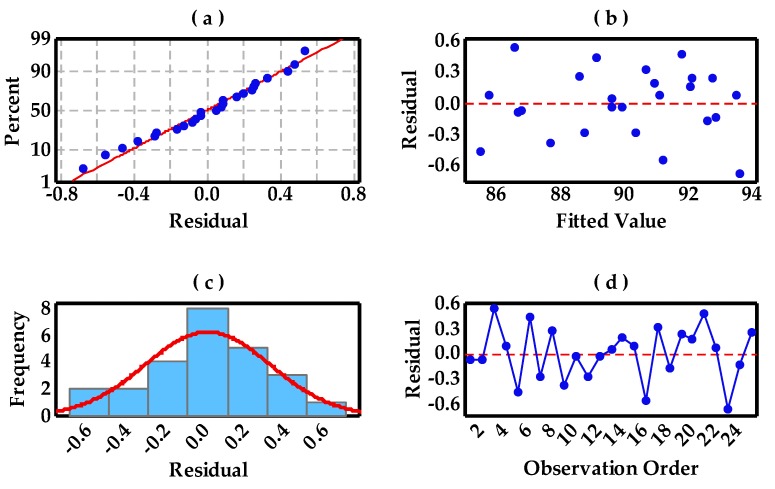
The residual plots for RD: (**a**) normal probability plot of residuals; (**b**) residuals versus fitted values; (**c**) histogram of the residuals; and (**d**) residuals versus order of data.

**Figure 9:**
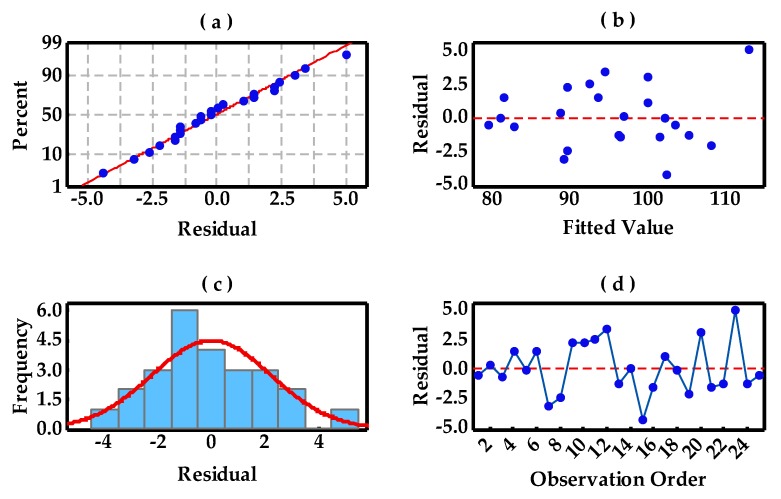
The residual plots for Vickers hardness: (**a**) normal probability plot of residuals; (**b**) residuals versus fitted values; (**c**) histogram of the residuals; and (**d**) residuals versus order of data.

Residual plots for RD and Vickers hardness are presented in Figure 8 and Figure 9, which contain the normal probability plot of residuals, residuals versus fitted values, the histogram of the residuals, and residuals versus the order of data, respectively. The points in Figure 8a form a straight line, meaning that the residuals of measured RD values with predicted values are normally distributed. Figure 8c intuitively shows a normal distribution pattern. A random distribution of residuals in Figure 8b suggests that this experimental model tends to yield the random error instead of an outlier. In addition, Figure 8d is a plot of all residuals in the order that the experimental results were collected and can be used to identify the non-random error and, here, it displays a beneficial experimental process control based on the SPC control chart standard. However, besides the similarity of normal distribution in Figure 9, the differences in these plots are concerned. The residual range (−5 to 5) becomes wider than that (−0.6 to 0.6) in Figure 8 and individual outliers may be produced, as shown in Figure 9b,c, which can be ascribed to the resultant porosity in these specimens, leading to the non-random error.

### 3.4. Forming Properties

**Figure 10:**
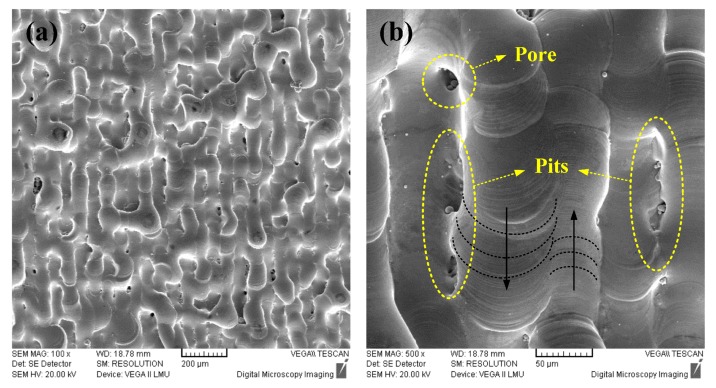
SEM surface morphologies of the Cu-4Sn with the RD 93.68%: (**a**) At a low magnification; and (**b**) At a high magnification.

Figure 10 shows the surface morphology of a Cu-4Sn specimen with 93.68% RD value in the Greco-Latin square design experiment. It is obvious that high surface roughness and discontinuous single tracks can be observed in Figure 10a. A number of defects, such as pores and pits (overlapping defects) are produced, contributing to the relatively low RD. The reasons for these defects can be ascribed to the inadequate laser energy, therefore, resulting in insufficient liquid phase and simultaneously causing a significant shrinkage of the molten pool due to the high surface tension and large viscosity of the liquid phase along with the resultant Marangoni flow. Additionally, it is responsible for a non-uniform deposition in the previous layer on account of characteristically rapidly solidifying with a limitation of spreading the molten pool in the overlapping track-track position (as shown in Figure 10b). As a consequence, according to the origins of these defects and above ANOVA, the densities of Cu-4Sn parts manufactured by SLM can be improved further through tailoring a higher energy intensity with a high LP while decreasing SS or narrowing HS are unfavorable methods since they tend to form the unstable molten pool (as shown in Figure 4) and seriously influence the production efficiency in the industrial application. The scaly image on the single tracks can contribute to identifying the scanning direction, as shown by the arrow in Figure 10b.

**Figure 11:**
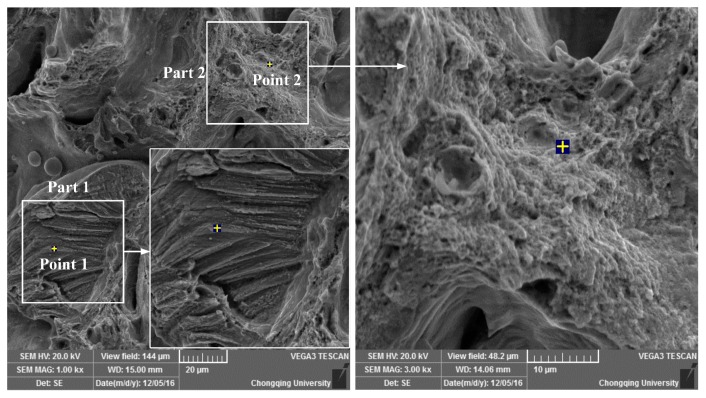
SEM micrograph of fracture surfaces during tensile testing.

The tensile specimens of Cu-4Sn were fabricated using relatively optimal parameters set in the above experiments. An attained ultimate tensile strength (UTS) 316–320 MPa is inferior to that of traditional pressure-processed Cu-4Sn (not less than 410 MPa). Figure 11 shows the fracture surfaces of these tensile samples in order to further understand the fracture mechanism. These samples simultaneously display two features in the Figure 11: step surfaces (Part 1) and dimples (Part 2), thus implying two fracture mechanisms (i.e., brittle cleavage fracture and ductile fracture). The production of two different fracture mechanisms can be ascribed to the segregation of tin in the sample (Figure 11) where point 1 of the step surfaces holds the same tin level as raw materials, while copper content is extremely high on point 2 of the dimples, as presented in Table 4. Some pores and grooves are also observed in these specimens, which can explain the low UTS. Generally, these defects are crack initiators and influence the overall structural performance during tensile testing. Therefore, defects should be reduced for the further improvement of UTS to meet the needs of applications.

## 4. Conclusions

Relatively optimal SLM processing parameters of Cu-4Sn based on the EOSINT M280 equipment were successfully identified and bulk specimens successfully manufactured for the first time using a systematic experimental approach. This proposed experimental approach consists of a single-track experiment and Greco-Latin square design experiment. The obtained conclusions are as follows:

1. The best RD value (93.68%) was obtained using an optimal SLM processing parametric set of LP = 195 W, SS = 50 mm/s and HS = 60 μm. The best Vickers hardness (118 HV 0.3/5) was also acquired using an SLM processing parametric set (i.e., LP = 195 W, SS = 200 mm/s, and HS = 120 μm).
2. During the experimental process, single-track experiments with different combinations of processing parameters can effectively narrow down selection windows for various parameters. Greco-Latin square design experiments with orthogonal parameter arrays can effectively reduce the number of experiments to acquire optimal processing parameters.
3. The ANOVA permits one to identify statistical influences of processing parameters on RD and Vickers hardness. It is noted that RD and Vickers hardness of Cu-4Sn specimens produced by SLM depend strongly on the LP.
4. The highest tensile strength achieved for Cu-4Sn in SLM is only 316–320 MPa, which is much less than that of traditional pressure-processed Cu-4Sn. The main reason for this is due to some internal defects of specimens, such as grooves, pores, and balling. These defects can be inhibited effectively by further improving LP.

## Figures and Tables

**Table 1:** SLM processing parameters used for single-track experiment.

Parameters	Value	Increment
LED	100–1000 J/m	100 J/m
LP	50–195 W	25 W
SS	0.05–1.95 m/s	-
Layer thickness	20 μm	-
Preheating temperature	80 °C	-
Substrate material	Die Steel (MS1)	-
Atmosphere	Argon (Oxygen level < 0.1%)	-

**Table 2:** Experiment design matrix and results of the Greco-Latin square design experiment.

Standard Order	Run Order	Factors			Responses		
		LP (W)	SS (mm/s)	HS (μm)	Density (g/cm^3^)	RD (%)	Vickers Hardness (HV 0.3/5)
1	6	100	100	100	7.577	86.71	79
2	5	100	150	120	8.282	86.61	89
3	19	100	200	140	7.657	87.10	82
4	3	100	250	160	8.211	85.84	83
5	1	100	300	180	7.717	85.04	81
6	20	125	100	120	7.728	89.57	95
7	13	125	150	140	8.296	88.51	86
8	15	125	200	160	7.991	88.85	87
9	18	125	250	180	8.284	87.29	92
10	8	125	300	100	8.010	89.59	92
11	24	150	100	140	8.000	90.09	95
12	22	150	150	160	8.135	89.92	98
13	11	150	200	180	7.820	89.69	95
14	23	150	250	100	8.213	91.12	97
15	12	150	300	120	7.925	91.20	98
16	25	175	100	160	8.257	90.68	95
17	10	175	150	180	8.244	91.03	101
18	17	175	200	100	7.786	92.42	102
19	16	175	250	120	7.769	92.37	106
20	4	175	300	140	7.943	92.22	103
21	14	195	100	180	8.347	92.41	100
22	21	195	150	100	7.966	93.68	104
23	2	195	200	120	8.128	93.04	118
24	9	195	250	140	8.017	92.87	104
25	7	195	300	160	8.049	93.10	103

**Table 3:** General linear model factors and results of ANOVA.

Factors Information					
Factor	Type		Levels	Values	
LP (W)	Fixed		5	100, 125, 150, 175, 195	
SS (mm/s)	Fixed		5	100, 150, 200, 250, 300	
HS (μm)	Fixed		5	100, 120, 140, 160, 180	
**RD ANOVA**					
**Source**	**DF**	**Adj SS**	**Adj MS**	**F-Value**	***p*-Value**
LP (W)	4	139.236	34.8090	170.59	0.000
SS (mm/s)	4	0.599	0.1499	0.73	0.586
HS (μm)	4	8.767	2.1918	10.74	0.001
Error	12	2.449	0.2041	-	-
Total	24	151.051	-	-	-
**Vickers Hardness ANOVA**					
**Source**	**DF**	**Adj SS**	**Adj MS**	**F-Value**	***p*-Value**
LP (W)	4	1646.8	411.7	41.31	0.000
SS (mm/s)	4	48.8	12.2	1.22	0.351
HS (μm)	4	216.8	54.2	5.44	0.010
Error	12	119.6	9.967	-	-
Total	24	2032	-	-	-

**Table 4:** The chemical compositions of different point on the fracture surface.

Elements	Weight Percentage (wt %)	
	Point 1	Point 2
Cu	95.33	100
Sn	4.67	0
Total	100	100
